# Supplementary material for: Analysis of the Function of the Lymphocytic Choriomeningitis Virus S Segment Untranslated Region on Growth Capacity In Vitro and on Virulence In Vivo
Source: Viruses. 2020 Aug 16;12(8):896. doi: 10.3390/v12080896 (PMC7474432; doi:10.3390/v12080896)
Supplement: Supplementary file 1 [file viruses-12-00896-s001.zip › Figure S1.pdf]

Figure. S1

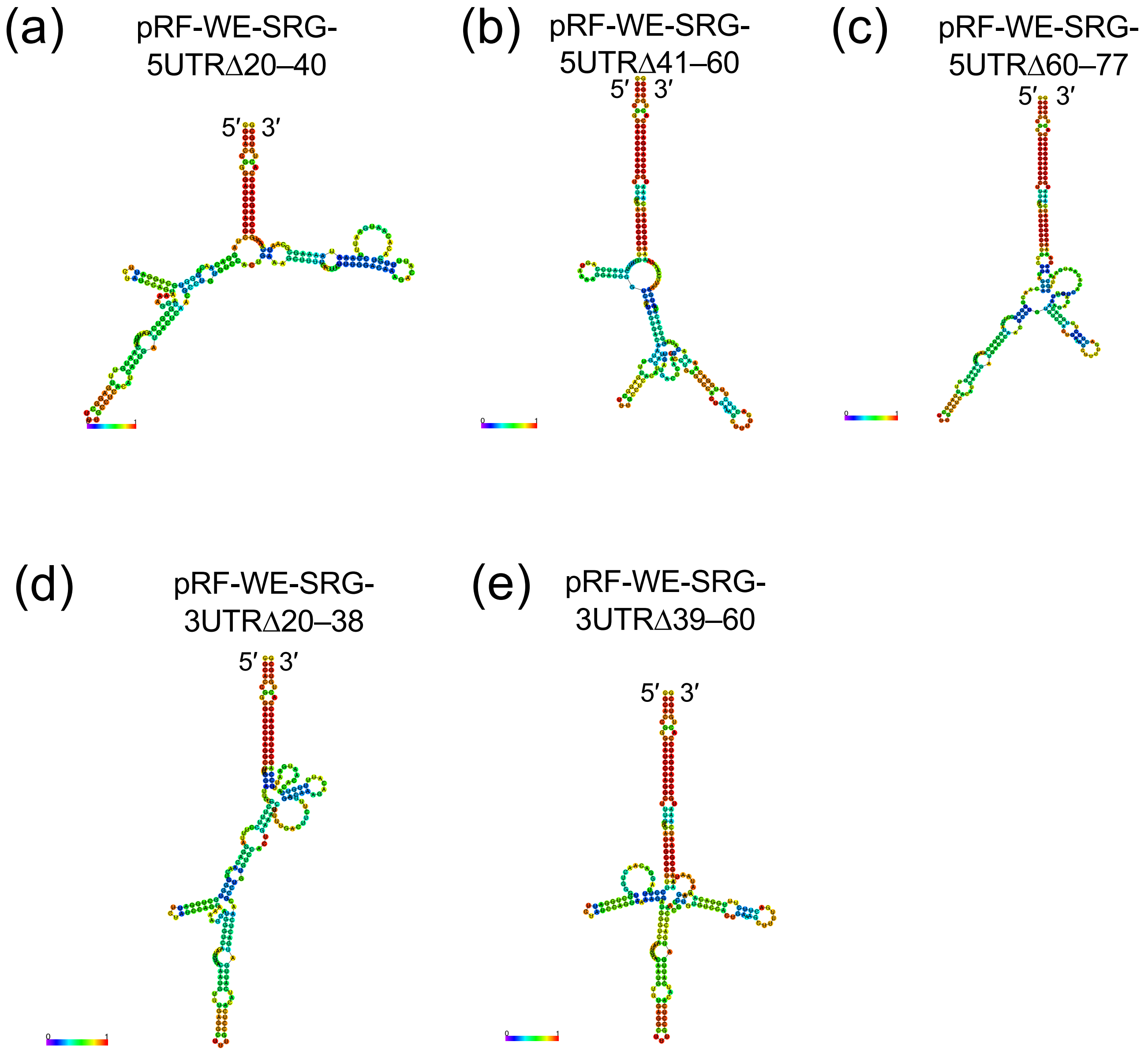

Figure S1. Prediction of RNA secondary structures of mutated LCMV strain WE (LCMV-WE) S segment UTRs which have 18–22 nt deletions in the 5′ or 3′ termini. (a to e). Predicted RNA secondary structures of LCMV-WE S segment UTRs which have 18–22 nt deletions in the 5′- or 3′-terminal UTRs. These RNAs were derived from (a) pRF-WE-SRG-5UTRΔ20–40, (b) pRF-WE-SRG-5UTRΔ41–60, (c) pRF-WE-SRG-5UTRΔ60–77, (d) pRF-WE-SRG-3UTRΔ20–38, and (e) pRF-WE-SRG-3UTRΔ39–60. RNA sequences of LCMV-WE S segment genome 5′-terminal and 3′-terminal UTRs with various mutation and 50 nt of ORF regional RNA sequences that were directly downstream of the UTRs were linked, sent to the CENTROIDFOLD server, and analyzed using the CONTRAfold model (weight of base pairs: 2^2). Each predicted base pair is colored with heat-color gradation from blue to red, corresponding to the base-pairing probability from 0 to 1. “5′” and “3′” indicate 5′ terminus and 3′ terminus of RNA, respectively. Detailed information about the various mutated pRF-WE-SRGs is given in Table 1, 2 and Figure 1.
