# Supplementary material for: Analysis of the Function of the Lymphocytic Choriomeningitis Virus S Segment Untranslated Region on Growth Capacity In Vitro and on Virulence In Vivo
Source: Viruses. 2020 Aug 16;12(8):896. doi: 10.3390/v12080896 (PMC7474432; doi:10.3390/v12080896)
Supplement: Supplementary file 1 [file viruses-12-00896-s001.zip › Figure S3.pdf]

Figure. S3

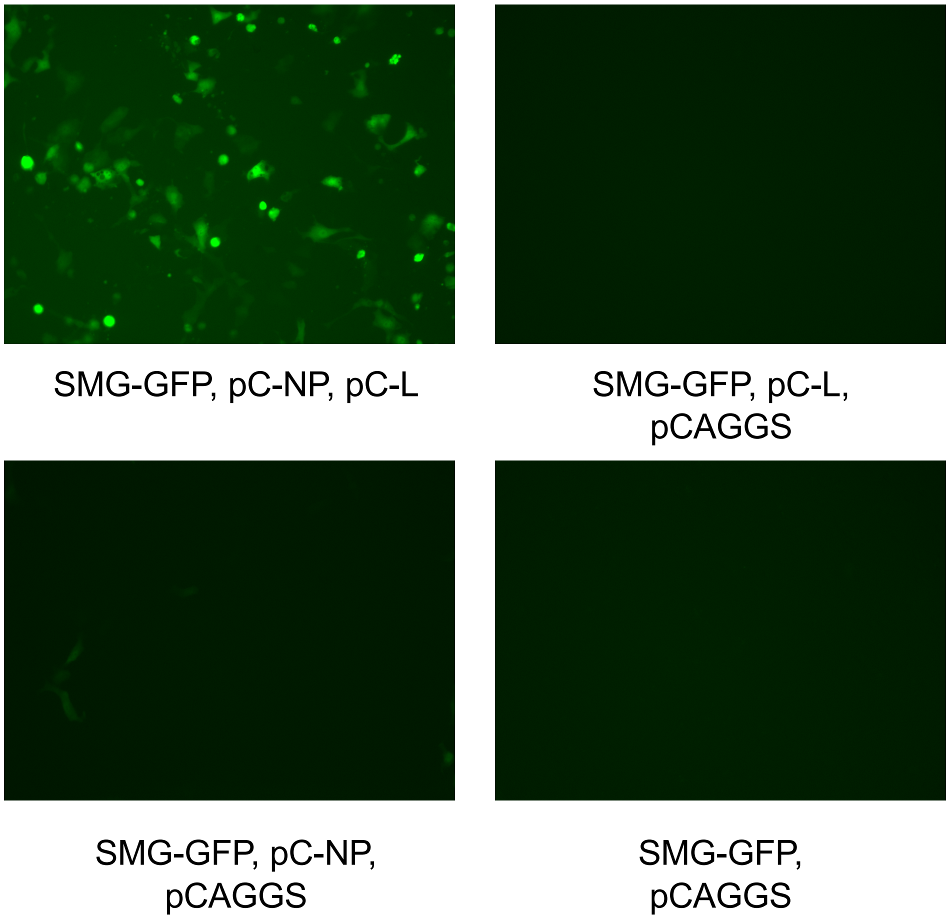

Figure S3. Establishment of a GFP expressing minigenome system for LCMV strain WE (LCMV-WE). BHK-21 cells were transfected with minigenome plasmids SMG-GFP, either pC-NP or pCAGGS, and either pC-L or PCAGGS. The transfected cells were incubated for 2 days at 37°C, and then the level of GFP expression was observed under a fluorescent microscope.
