# Supplementary material for: Analysis of the Function of the Lymphocytic Choriomeningitis Virus S Segment Untranslated Region on Growth Capacity In Vitro and on Virulence In Vivo
Source: Viruses. 2020 Aug 16;12(8):896. doi: 10.3390/v12080896 (PMC7474432; doi:10.3390/v12080896)
Supplement: Supplementary file 1 [file viruses-12-00896-s001.zip › Figure S4.pdf]

Figure. S4

(a)

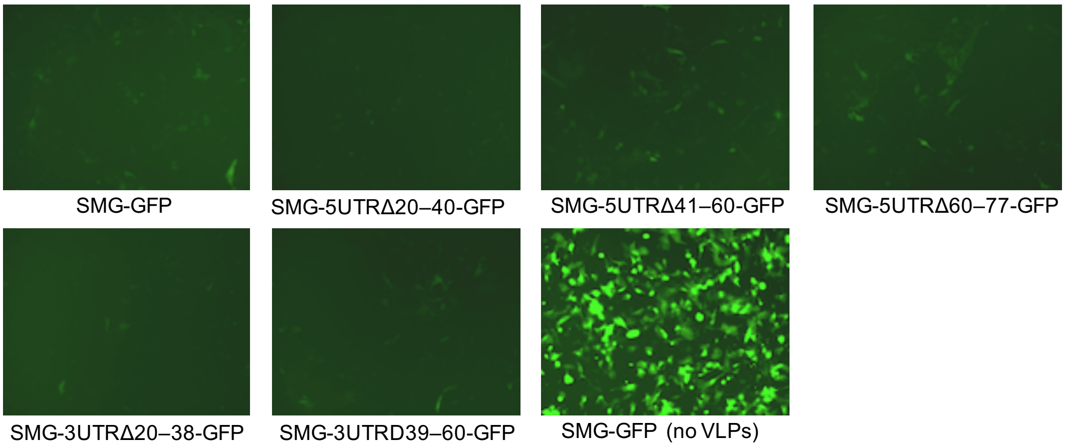

(b)

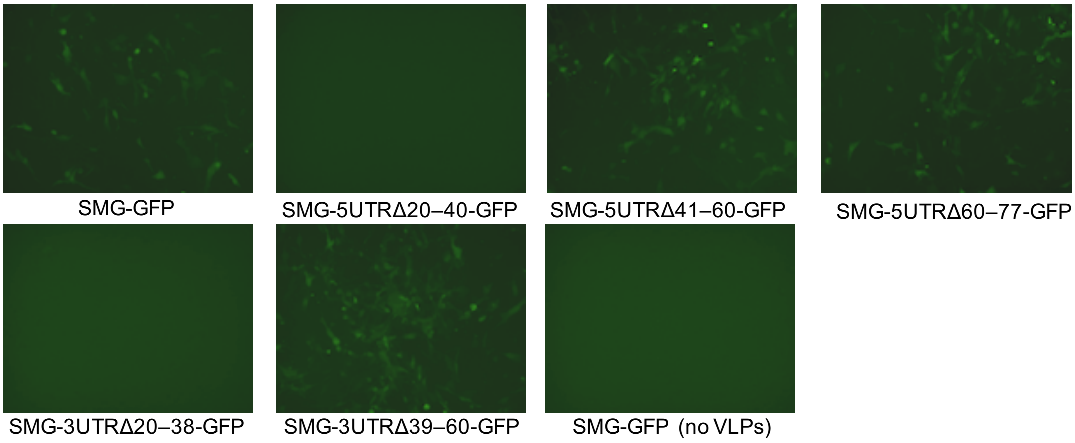

Figure S4. Evaluation of the efficiency of viral genome transcription, replication, and packaging in virus-like particles (VLPs). (a) Viral genome transcription and replication were assessed in BHK-21 cells transfected with SMG-GFP, SMG-5UTRΔ20–40-GFP, SMG-5UTRΔ41–60-GFP, SMG-5UTRΔ60–77-GFP, SMG-3UTRΔ20–38-GFP or SMG-3UTRΔ39–60-GFP under conditions of the co-expression of NP, L protein, GPC, and Z protein. BHK-21 cells were transfected with each mutated SMG-GFP, pC-NP, pC-L, pC-Z, and pC-GPC. As a background control for the rescue of LCMV RNA analogs into VLPs, cells transfected with SMG-GFP, pC-NP, and pC-L were used [SMG-GFP (no VLPs)]. After incubation for 48 hours at 37°C with 5% CO_2, GFP expression was examined. (b) Packaging of viral genome RNA analogs derived from SMG-GFP, SMG-5UTRΔ20–40-GFP, SMG-5UTRΔ41–60-GFP, SMG-5UTRΔ60–77-GFP or SMG-3UTRΔ39–60-GFP into VLPs were assessed. Supernatants from each well of Figure S4a were harvested and used to infect fresh monolayers of BHK-21 cells, which were then incubated for 4 hours at 37°C before adding helper LCMV. Ninety hours post-infection, the passage culture was examined for GFP expression.
